# A Site-Selection Strategy Based on Polarity Sensitivity for Cochlear Implants: Effects on Spectro-Temporal Resolution and Speech Perception

**DOI:** 10.1007/s10162-019-00724-4

**Published:** 2019-06-03

**Authors:** Tobias Goehring, Alan Archer-Boyd, John M. Deeks, Julie G. Arenberg, Robert P. Carlyon

**Affiliations:** 1 Medical Research Council Cognition and Brain Sciences Unit, University of Cambridge, 15 Chaucer Road, Cambridge, CB2 7EF UK; 2 Department of Speech and Hearing Sciences, University of Washington, 1417 NE 42nd St., Seattle, WA 98105 USA

**Keywords:** cochlear implants, speech perception, detection thresholds, channel selection

## Abstract

Thresholds of asymmetric pulses presented to cochlear implant (CI) listeners depend on polarity in a way that differs across subjects and electrodes. It has been suggested that lower thresholds for cathodic-dominant compared to anodic-dominant pulses reflect good local neural health. We evaluated the hypothesis that this polarity effect (PE) can be used in a site-selection strategy to improve speech perception and spectro-temporal resolution. Detection thresholds were measured in eight users of Advanced Bionics CIs for 80-pps, triphasic, monopolar pulse trains where the central high-amplitude phase was either anodic or cathodic. Two experimental MAPs were then generated for each subject by deactivating the five electrodes with either the highest or the lowest PE magnitudes (cathodic minus anodic threshold). Performance with the two experimental MAPs was evaluated using two spectro-temporal tests (Spectro-Temporal Ripple for Investigating Processor EffectivenesS (STRIPES; Archer-Boyd et al. in J Acoust Soc Am 144:2983–2997, 2018) and Spectral-Temporally Modulated Ripple Test (SMRT; Aronoff and Landsberger in J Acoust Soc Am 134:EL217–EL222, 2013)) and with speech recognition in quiet and in noise. Performance was also measured with an experimental MAP that used all electrodes, similar to the subjects’ clinical MAP. The PE varied strongly across subjects and electrodes, with substantial magnitudes relative to the electrical dynamic range. There were no significant differences in performance between the three MAPs at group level, but there were significant effects at subject level—not all of which were in the hypothesized direction—consistent with previous reports of a large variability in CI users’ performance and in the potential benefit of site-selection strategies. The STRIPES but not the SMRT test successfully predicted which strategy produced the best speech-in-noise performance on a subject-by-subject basis. The average PE across electrodes correlated significantly with subject age, duration of deafness, and speech perception scores, consistent with a relationship between PE and neural health. These findings motivate further investigations into site-specific measures of neural health and their application to CI processing strategies.

## INTRODUCTION

Cochlear implants (CIs) allow many users to understand speech well in quiet acoustic situations. However, there is a large variability in the performance between users and even the most successful show much worse speech perception in background noise compared to normal-hearing listeners (Friesen et al. 2001; Cullington and Zeng 2008). Efforts to develop noise reduction techniques for improved speech recognition in background noise with CIs have shown promising results but still remain an area of active research and do not eliminate the large differences in outcomes among CI users (Hu and Loizou 2010; Dawson et al. 2011; Goehring et al. 2017). Possible underlying reasons for the speech perception difficulties and variable outcome in CI listeners include the user-specific pattern of neural survival (Khan et al. 2005; Fayad and Linthicum 2006), the broad spread of neural activation along the auditory nerve (at least in the monopolar mode used clinically (Shannon 1983; Hughes and Stille 2010)), and the variability due to surgical trauma and electrode placement in the cochlea (Finley and Skinner 2008; Carlson et al. 2011). Together, these effects contribute to reduced spectro-temporal resolution and to distortions of the frequency-to-place mapping of sound in the cochlea. This in turn is likely to impair speech perception especially in noisy listening situations with background sounds (Friesen et al. 2001; Fu and Nogaki 2005).

Several studies have proposed electrode-deactivation strategies as a means to improve speech-in-noise perception in CI users. The criteria used for (de-) activation have been based on measures of electrode discrimination (Zwolan et al. 1997; Saleh et al. 2013; Vickers et al. 2016), modulation detection thresholds (Zhou and Pfingst 2012; Garadat et al. 2012, 2013), stimulus detection thresholds (Bierer and Litvak 2016; Zhou 2017), and the results of CT-imaging techniques (Noble et al. 2013, 2014). Reducing the number of stimulation sites may improve spectral resolution by decreasing channel interactions and can in principle be used to selectively deliver electrical stimulation to better-functioning neural regions along the cochlea. Indeed, two studies have shown that electrode discrimination scores can successfully be used to deactivate electrodes from the everyday MAP of CI users to improve speech perception (Zwolan et al. 1997; Saleh et al. 2013), whereas another study based on electrode discrimination did not find such differences in performance (Vickers et al. 2016). Spectral resolution and speech recognition in quiet and in noise have been improved relative to the clinical MAP by using a site-selection strategy based on low-rate (80 pps) detection thresholds, which were proposed to reflect neural health (Zhou 2016, 2017), while no group effects were found in a study that used high-rate (997 pps) detection thresholds for selecting deactivation sites (Bierer and Litvak 2016). Improvements in speech-in-noise perception, relative to the clinical MAP, have been obtained using strategies based on modulation detection thresholds (Garadat et al. 2013) and CT-imaging techniques (Noble et al. 2013, 2014).

Although site-selection strategies may preferentially stimulate those electrodes that most effectively convey information needed to understand speech, there is a potential disadvantage of reducing the number of stimulated channels. While previous studies have shown that speech perception does not improve beyond about 4 to 10 spectral channels of information (Dorman et al. 1997; Friesen et al. 2001; Garnham et al. 2002; Fu and Nogaki 2005)—fewer than the number of electrodes in modern CIs—two recent studies have shown that speech recognition performance in noise can improve as the number of channels is increased above 8 (Schvartz-Leyzac et al. 2017) or 12 (Croghan et al. 2017). Those studies suggest that deactivating some electrodes could degrade performance even when a fairly large number of electrodes remain activated. However, the performance improvements with more than 8 or 12 active electrode channels observed by Croghan et al. and by Schvartz-Leyzac et al. were small, and the advantages with more active electrode channels may have been due to the increased similarity of the experimental MAPs to the everyday MAP of the subjects or to the fact that the reduced-channel MAPs’ performance was decreased by selecting a set of relatively poorer electrode sites. In general, there is growing evidence that site-selection strategies can potentially improve speech-in-noise perception in some CI users when a small proportion of electrodes is deactivated based on individualized measures of neural function and/or electrode position at each electrode site. However, the potential benefits of selectively stimulating “more effective” channels must always be weighed against the potential disadvantages in reducing the number of channels of information.

For a site-selection method to be successfully implemented in CI speech processors, it is desirable to have a reliable measure of the electrode-nerve interface and of the functioning of neural processes at each electrode site (Bierer and Faulkner 2010; Pfingst et al. 2015). Understanding the neural bases of any effect is likely to prove important for understanding why a manipulation does not work, or works only in some subjects, and in the development of new and more effective methods. Although CT image-guided approaches provide a high level of information about the placement of the electrode array within the cochlea (Noble et al. 2013, 2014; Long et al. 2014; DeVries et al. 2016), they may not be available for many CI users due to the health risk from radiation exposure and do not provide information on neural survival. In contrast, single-electrode psychophysical measures can be safely obtained with any CI user able to participate in the task. Several studies have reported that psychophysical measures of single-electrode detection thresholds show substantial across-listener and across-electrode variability, and it has been suggested that this variability may be used for estimating the individual pattern of neural functioning along the electrode array (Pfingst et al. 2004; Bierer and Faulkner 2010; Bierer et al. 2015a; Cosentino et al. 2016; Mesnildrey 2017; Carlyon et al. 2018). Such measures have been applied in site-selection strategies, and improvements in speech perception compared to the everyday MAPs were observed. Those advantages were observed for some subjects, but not at group level, when electrodes were selected and deselected on the basis of high-rate thresholds (Bierer and Litvak 2016) and for all subjects and at group-level when (de-) selection was based on low-rate thresholds (Zhou 2016, 2017). The improvements reported by Zhou (2016, 2017) were obtained even without allowing subjects to acclimatize to the experimental settings beforehand. Here, we propose an estimate of local neural health using the difference between low-rate detection thresholds for trains of asymmetric pulses of opposite polarity and evaluate its potential applicability in a site-selection strategy for CI users.

Studies using animal models have found greater sensitivity to cathodic stimulation than to anodic stimulation (Hartmann et al. 1984; Miller et al. 1999; Miller et al. 2004). The reverse is true for human CI users, who require less current in anodic than cathodic stimulation mode to obtain comfortable listening levels (Macherey et al. 2006, 2008; Van Wieringen et al. 2008) or electrically evoked responses (Undurraga et al. 2010; Undurraga et al. 2013; Spitzer and Hughes 2017; Hughes et al. 2018). A potential reason for the difference between human and animal data comes from computational studies that modeled the effect of degenerated peripheral processes of the spiral ganglion cells, compared to cells with intact peripheral processes (Rattay 1999; Rattay et al. 2001). Thresholds were increased in all cases with degenerated peripheral processes but more so for cathodic than for anodic stimulation and especially for the human model compared to the animal model. These predictions for the human model show that the ratio between anodic and cathodic thresholds depends strongly on the survival of peripheral processes, with lower cathodic than anodic thresholds for regions with more intact peripheral processes and lower anodic than cathodic thresholds for regions with more degenerated peripheral processes (cf. Resnick et al. 2018). This observation is consistent with the difference in polarity sensitivity found between acutely deafened animal models (Hartmann et al. 1984; Miller et al. 1999, 2004), which retain intact peripheral processes for up to 2 months after inducing hearing loss (Leake and Hradek 1988), compared to human CI users (Macherey et al. 2008; Macherey et al. 2017; Carlyon et al. 2018) that have been deaf for longer periods of time and thus tend to have more degenerated peripheral processes (Johnsson et al. 1981; Zimmermann et al. 1995; Fayad and Linthicum 2006).

While previous studies found anodic stimulation to be more efficient in human CI users at supra-threshold stimulation levels (Macherey et al. 2008; Undurraga et al. 2010; Spitzer and Hughes 2017), recent results indicate that polarity sensitivity at threshold is subject-dependent and varies across electrodes for a given CI user (Macherey et al. 2017; Mesnildrey 2017; Carlyon et al. 2018; Hughes et al. 2018). Consequently, it might be possible to use the subject-specific pattern of polarity sensitivity to estimate the presence of intact or degenerated peripheral processes and to serve as an indicator of the local neural health along the electrode array. The polarity sensitivity at threshold level, PE, is obtained from a population of spiral ganglion cells with potentially varying degrees of degeneration of the peripheral processes and therefore expected to be of gradual nature. This measure is based on detection thresholds that can be obtained with lower stimulation current than used for supra-threshold measures, thereby potentially improving its spatial selectivity. Furthermore, the computation of a difference metric between thresholds in both polarities at each electrode site normalizes somewhat for the distance of the electrode array with respect to the targeted spiral ganglion nerve cells. Indeed, Mesnildrey (2017) measured the PE from multiple electrodes in nine subjects from whom they had postoperative CT scans and reported that the PE did not correlate with the distance from the spiral ganglion cells, as estimated from the electrode-modiolar distance. An additional advantage of the PE measure is that, because it is a difference score, it is unlikely to be affected by cognitive differences between subjects (Carlyon et al. 2018).

We measured detection thresholds in a group of CI users for anodic- and cathodic-dominant triphasic pulse trains and calculated the polarity sensitivity as the difference between the two thresholds (cathodic minus anodic) at all active sites along the electrode array for each subject. The pattern of polarity sensitivity was then used in a site-selection strategy to generate two experimental MAPs for each subject based on their clinical MAP, one with the five “best” sites (those with the smallest PEs) deactivated and one with the five “worst” sites (largest PEs) deactivated while never allowing three adjacent sites to be deactivated. Subjects completed a set of listening tests to evaluate their performance in terms of spectro-temporal resolution and speech perception in quiet and in background noise with the experimental MAPs and with a MAP that used all electrodes, similar to their clinical MAP.

The main goal was to investigate the reliability of the polarity sensitivity measure on an individual scale and to evaluate its potential use in guiding a site-selection strategy for improving speech perception in CI users. Furthermore, we analyzed potential relationships between the estimated neural health and speech performance across users, based on the hypothesis that subjects with better neural health will also be more successful in utilizing their CI. Finally, we explored whether spectro-temporal tests can be used to reliably predict speech perception benefits between experimental conditions on an individual basis, in order to facilitate the evaluation of optimized programming strategies in clinical environments.

## METHODS

### Subjects

Eight post- or peri-lingually deafened, native speakers of British English took part. Their mean age was 62 years, with a range from 48 to 72 years. Subjects were unilaterally implanted users of an Advanced Bionics (“AB”; Valencia, CA, USA) HiRes 90K™ cochlear implant and had more than 2 years of experience with their device with a mean duration of implant use of 5.5 years. Half of the subjects were implanted with a pre-curved, mid-scalar electrode (HiFocus™ Mid-Scala, MS) and the other half with a straight lateral wall electrode (HiFocus™ 1J, 1J). Only the implanted ear of each subject was used for the presentation of stimuli; if a subject was wearing a hearing aid in the other ear, then it was turned off during the experiment. Prior to the experiment, the most recent clinical MAP was obtained for each subject (with usage experience with the clinical MAPs ranging from 10 months to 2 years). Details about the demographic information and devices used by the eight subjects are given in Table 1.

**Table 1:** Demographic information and details about CI devices used by the subjects

Participant	Specifier	Sex	Age (years)	Duration implanted (years)	Duration of deafness (years)	Etiology/acquired pre-/post-lingual	CI speech processor	CI electrode array	Pulse width (us) in proc. strategy	Electrodes deactivated
S1	AB3	M	71	10	35	Otosclerosis/post-ling. progression	HR90K	HiFocus 1J	30.5	EL12
S2	AB1	M	72	9	40	Unknown/post-ling.	HR90K	HiFocus 1J	26.0	EL16
S3	AB6	F	69	5	64	Unknown/peri-ling.	HR90K	HiFocus 1J	35.0	EL16
S4	AB24	F	48	2	3	Unknown/post-ling.	HR90K Advantage	HiFocus MS	34.1	EL16
S5	AB26	F	56	4	20	Unknown/post-ling.	HR90K Advantage	HiFocus MS	23.3	None
S6	AB23	F	59	2	57	Enlarged vestibular aqueduct/post-ling.	HR90K Advantage	HiFocus MS	22.4	None
S7	AB25	F	64	2	33	Sinus infection/post-ling.	HR90K Advantage	HiFocus MS	18.0	EL16
S8	AB2	F	57	10	26	Possible otoxicity/post-ling.	HR90K	HiFocus 1J	35.0	EL16

The study was part of a larger research program that was approved by the National Research Ethics committee for the East of England. Before commencing, subjects gave their informed consent and were informed that they could withdraw from the study at any point. Subjects were paid for taking part and reimbursed for travel expenses.

### Technical Equipment and Software

All experiments were performed using a battery-powered laptop computer running Microsoft Windows 10 Pro (Dell XPS 15, model 2017). Experimental sessions took place in a quiet testing room located in the MRC Cognition and Brain Sciences Unit at the University of Cambridge. The experimenter and the subject were sitting at a desk while one of them used the laptop computer, depending on the stage of the experiment underway at the time.

A direct-stimulation experiment was performed for measuring the psychophysical detection thresholds (described in part IIc). The technical setup for this part of the experiment consisted of an AB Clinical Programming Interface (CPI) connected to an AB Platinum Sound Processor (PSP), which was in turn controlled using BEDCS software (Ver. 1.18.337; Advanced Bionics, Valencia, CA, USA) with experimental programs written in MATLAB (Ver. 2014a; The Mathworks, Nattick, MA, US). The research processor was connected to the laptop with a USB-to-serial port converter and delivered the stimuli directly to the CI of the subjects via a cable and RF transmitter coil provided by AB. Stimuli were controlled by the experimental software to exceed neither the electrical compliance limit (7 V) of the research processor nor the safety charge limit of the electrode array. For each subject, impedance measures were performed for each electrode at the beginning of every testing session using AB’s Soundwave software (Ver. 2.3) to calculate maximum current levels within compliance limits. Following standard practice in our laboratory, impedances were checked at the end of all sessions that used this direct-stimulation method.

The spectro-temporal and speech intelligibility tests (described in part IIf) did not involve direct stimulation but instead used a programmable Harmony speech processor (AB) that was battery-powered and worn by the subject during the listening tests. The stimuli were delivered to the subject using an external USB soundcard (Roland UA-55 Quad-Capture USB) that was connected to the auxiliary (AUX) input port of the processor with an audio-cable provided by AB and with the input from the microphone disabled. The utilization of a clinical AB speech processor for this part of the experiment ensured that the presentation of stimuli did not exceed compliance limits and comfortable listening levels as specified in the individual clinical MAP of the subject. The stimulus presentation level for one spectro-temporal test (Spectro-Temporal Ripple for Investigating Processor EffectivenesS (STRIPES), see part IIf) was set to most comfortable level using the default STRIPES test stimulus and by adjusting the manual volume control of the soundcard. For the other spectro-temporal test (Spectral-Temporally Modulated Ripple Test (SMRT), see part IIf) and the speech intelligibility tests, the presentation level was calibrated to 60 dB SPL using the direct-connect calibration procedure implemented in the AB research software (LIST Player Ver. 3, Advanced Bionics, Valencia, CA, USA) and by adjusting the manual volume control of the soundcard accordingly. For each subject and at the beginning of every test part that was performed for the experiment, the presentation levels were confirmed by the subjects to be comfortable to them.

### Psychophysical Detection Thresholds

The goal of this part of the experiment was to detect individual differences in polarity sensitivity at threshold level across the electrode array for each subject, so as to determine an estimate of local neural health. The stimuli for the measurement of detection thresholds in anodic- and cathodic-dominant polarities consisted of monopolar, triphasic stimuli for which the central phase had twice the amplitude of the first and last phases of the stimulus. The polarity of the central phase defined the polarity of the stimulus (anodic-cathodic-anodic, ACA or *cathodic*, and cathodic-anodic-cathodic, CAC or *anodic*). The duration of each of the three phases was 43.1 μs, and stimuli were presented at a rate of 80 pps and with a total stimulus duration of 300 ms. The current level was specified and controlled in microampere by the low-level direct-stimulation routines but was scaled to decibel values when set by the experimental software.

Before the measurement of detection thresholds, subjects completed loudness ratings for both ACA and CAC stimuli for each electrode activated in their clinical MAP (see Table 1). Electrical stimulation always started at zero current level and was increased in small current steps while obtaining feedback from the subjects on the perceived loudness by using a loudness chart (from step 1 “Just Noticeable” up to step 7 “Loud but Comfortable”) and tracking step 6 “Most Comfortable.” This procedure was necessary to obtain safe and comfortable initial stimulation levels for the following adaptive threshold measurements.

For the measurements of detection thresholds (THRs), an adaptive one-up/one-down tracking procedure was used. This was similar to a Békésy-tracking scheme applied independently to each electrode (i.e., there were no changes in stimulation electrode during an adaptive track). The initial presentation level was set between 70 and 98 % of the obtained MCL in current level (step 6 of the loudness chart) for that electrode and polarity combination. For electrodes with a comparatively larger dynamic range as indicated by the loudness ratings, a smaller percentage of the MCL was used as initial level to reduce the number of steps necessary to reach threshold level. Conversely, for electrodes with a smaller dynamic range, a higher percentage of the MCL was used as initial level to ensure that a sufficient number of trials were clearly audible before reaching the first reversal. This was done to reduce the time needed for the subject to complete the adaptive procedure while ensuring a stable adaptive track. Subjects pressed the space bar of the computer keyboard each time they heard a sound. When subjects responded to the stimuli within a time window of 3 s, the presentation level was decreased by one step size and a new stimulation was triggered after a randomly chosen delay of between 2 and 3 s. If subjects did not respond within 3 s after the stimulus presentation, the level was increased by one step size and presented after a randomly chosen delay of between 0.1 and 0.6 s. This resulted in a stimulus presentation every 2 to 6 s. The initial step size was 0.5 dB and was reduced to 0.2 dB after the first reversal (with a minimum step size of 4 μA imposed by the direct-stimulation routines). The adaptive procedure stopped after eight reversals and the THR level was estimated as the average of the stimulus levels at the last six reversal points.

The presentation order of electrodes was randomized per subject and two adaptive tracks were performed for each threshold estimate. In the first run, stimuli were presented at every electrode in randomized order for both polarities (ACA and CAC), randomly choosing which polarity was presented first. In the second run, electrodes were presented in reversed order, and also the polarity was chosen in reversed order to the first run to control for order effects of the presentation. The average of the two runs was taken as the final THR estimate for each electrode-polarity combination. In total, this procedure took about 2 h and required up to 64 adaptive tracks to be completed by the subjects (with a maximum of 16 electrodes for the two polarities and two runs).

### Site Selection Based on Polarity Sensitivity

The polarity effect (PE) was defined as the difference in sensitivity to CAC versus ACA stimuli at threshold level. It was calculated for each subject and each electrode by converting the obtained THR levels to decibels and subtracting the anodic from the cathodic thresholds. A negative, or small, PE value reflects greater sensitivity to cathodic than to anodic stimulation and is hypothesized to indicate a healthier neural region due to a higher proportion of intact peripheral processes of the excited neurons. In contrast, a positive, or large, PE value is hypothesized to indicate a neural region with poorer neural health due to a higher proportion of degenerated peripheral processes. This estimate of local neural health along the electrode array was used to guide a site-selection strategy for improving listening performance in CI users.

Three experimental MAPs were generated in Soundwave™ for each subject. For the first two experimental MAPs, the five electrodes with either the highest (MAP 1) or lowest (MAP 2) PE values were selected sequentially and then deactivated in the clinical MAP of that subject, with the constraint that three adjacent electrodes could not be deactivated. If, at any point during the construction of the experimental MAPs, the selection of the next deactivated electrode would have resulted in three adjacent electrodes being deactivated in the experimental MAP, then this electrode was kept active and the next highest or lowest electrode was selected that did not yield three adjacent electrodes to be turned off. This rule was imposed in order to avoid an extreme cluttering of deactivated electrodes in one region of the electrode array. No further selection restrictions were applied. The third experimental MAP (MAPC) served as a control condition, and the same electrodes were active as in the clinical MAP of each subject.

For all three experimental MAPs tested in the evaluation experiment, the coding strategy was changed to HiRes-S (roughly similar to continuous-interleaved-sampling, CIS, without any current focussing or steering), while keeping the same pulse duration as used with the coding strategy in the subjects’ clinical MAP (all subjects used HiRes-Optima in their clinical MAP, a strategy based on CIS with additional current steering). This led to an automatic adjustment of the channel stimulation rate for all three MAPs in the clinical software Soundwave™ to compensate for the change in loudness resulting from the change in coding strategy. When further switching off electrodes in the experimental MAPs (MAP 1 and MAP 2), the clinical software again automatically adjusted the channel stimulation rate to provide the same overall stimulation rate per cycle depending on the pulse width used by each subject (see Table 1). This resulted in a change in channel stimulation rate by the ratio of all active electrodes *M* divided by the remaining active electrodes in the experimental MAPs *M* − 5 (resulting in a factor of *M* / (*M* − 5) ~ 1.5). The input signal was changed to AUX ONLY to mute the microphone input and to automatically deactivate all further adaptive post-processing functions (for example, any noise reduction function that was active) and the internal telecoil was switched off to avoid potential interference. The threshold (*T*) and most comfortable (*M*) levels given by the clinical MAP were unchanged in the experimental MAPs for each electrode. The number of remaining active electrodes in the experimental MAPs (MAP 1 and MAP 2) led to an adjustment of the center frequencies and bandwidths used for the input analysis filter bank (see Table 2) but was the same in both MAPs for each subject. The allocation of input sound spectral information to stimulation electrode was changed depending on the location of the deactivated electrodes in the two MAPs per subject, to provide all input sound information to the active sites of stimulation for that MAP. The differences in terms of number of active electrodes, changes in spectral analysis filters, and channel stimulation rates were the same between the experimental MAPs (MAP 1 and MAP 2) in respect to MAPC, which served as a control condition most similar to the clinical MAP of the subjects.

**Table 2:** Center frequencies used for the experimental MAPs with 10, 15, or 16 active channels

MAP	Spectral channel															
	1	2	3	4	5	6	7	8	9	10	11	12	13	14	15	16
10 ch.	356	534	704	926	1220	1607	2117	2788	3673	6438						
15 ch.	336	463	556	668	804	965	1160	1394	1674	2012	2417	2904	3490	4193	6638	
16 ch.	333	455	540	642	762	906	1076	1278	1518	1803	2142	2544	3022	3590	4264	6665

In order to evaluate the possibility of loudness differences between the three MAPs under test, that may have affected listening performance (for example, speech intelligibility), subjects completed a loudness rating procedure for all three experimental MAPs for comparison purposes. A 2.5-s-long white noise signal was generated and shaped with the long-term average spectrum of 10 sentence lists of the speech material used for the listening experiments (see “Performance Evaluation” section) and calibrated to the same root-mean-square level as the speech stimuli. This signal was then used to perform loudness ratings with each experimental MAP and presented via the AUX port of the Harmony research processor. The playback started at a presentation level of − 40 dB relative to the presentation level used for the listening experiment and was then increased using a MATLAB script while obtaining feedback from the subjects on the AB loudness chart.

### Performance Evaluation

#### Spectro-Temporal Tasks

**Fig. 1:**
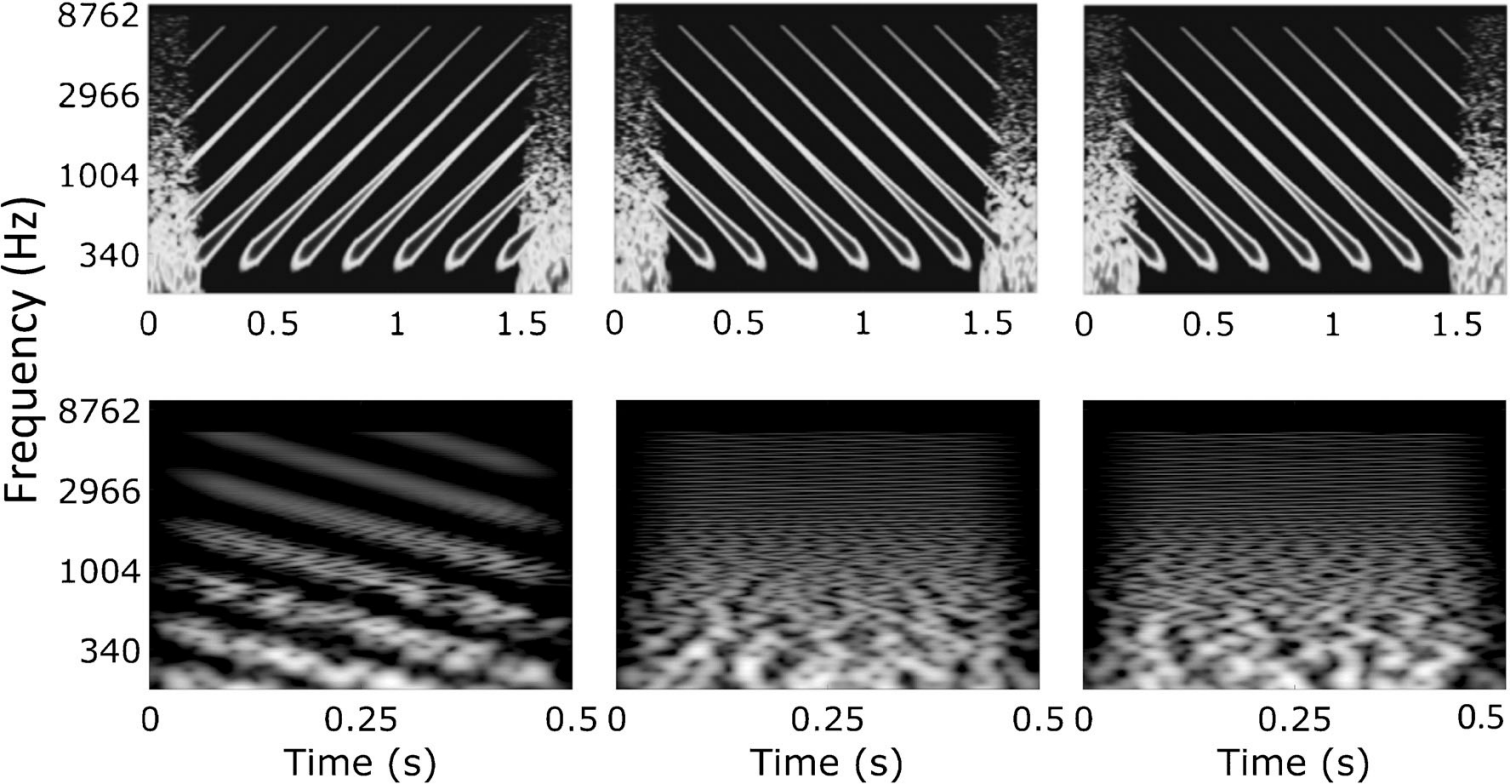
Time-frequency plots of STRIPES stimuli (upper panel, density of 5) and SMRT stimuli (lower panel, RPOs of 1 and 20) used for a single trial in the forced-choice task. The target stimulus is shown in the left position with the two reference stimuli shown in the middle and right positions

We used two spectro-temporal non-speech tasks. One of these, the STRIPES test was developed in our laboratory (Archer-Boyd et al. 2018). The test uses an adaptive procedure to measure the threshold at which the subject can just distinguish the target stimulus from two reference stimuli in a three-interval, two-alternative forced-choice task. Stimuli consisted of 1-s-long, concurrent exponential sine sweeps moving up or down in frequency from 250 to 8000 Hz. The subject had to select the target interval, which was either the first or last interval and which was always an upward sweep; the other two intervals contained downward sweeps (Fig. 1, top row). The number of concurrent frequency sweeps (the “density”) was varied to titrate difficulty, with the task being very easy at a density close to 1, and progressively harder at higher densities. The starting frequency was roved across trials and the beginning and end of each interval was masked by short noise bursts to reduce the salience of onset and offset cues. An adaptive two-up/one-down procedure started with a sweep density of 1.1 (equal to the total number of, but not necessarily uninterrupted, sweeps present during a 1-s interval) and adjusted the density per trial with a density step size of 0.5 (for the first four reversals) and 0.2 (for the last eight reversals). The test was complete after 12 reversals and the final score of the run was calculated as the average density of the last four reversals.

The other spectro-temporal measure was the SMRT test developed by Aronoff and Landsberger (2013). Stimuli were generated using a non-harmonic tone complex with 202 equal-amplitude pure-tone components from 100 to 6400 Hz that were modulated by a sine wave with a defined number of ripples per octave (RPO). The SMRT test involved a three-interval, forced-choice task, in which two of the three 500-ms-long intervals contained a reference stimulus with 20 RPO, and the other interval contained the target stimulus that was initialized with 0.5 RPO and adjusted using an one-up/one-down procedure with a step size of 0.2 RPO per trial. The phase of the ripple at the onset of the stimuli was pseudo-randomized and a test run was completed after ten reversals, of which the last six were used to calculate the final score by averaging. Software to perform the SMRT test was obtained from the official website provided by the developers (http://smrt.tigerspeech.com). The SMRT test was developed for hearing-impaired listeners and selected in this study to serve as a comparison condition to STRIPES because it is widely used with CI listeners and has been shown to correlate with their mean speech perception scores in quiet and in noise in previous studies (Holden et al. 2016; Lawler et al. 2017; Zhou 2017). However, for the SMRT test, CI listeners may perform the task based on cues different from spectro-temporal processing. For example, amplitude modulations within a single channel may be sufficient to distinguish the target from the reference stimulus at low RPOs (Archer-Boyd et al. 2018). This can be seen in the bottom part of Fig. 1, where at any one CF the amplitude fluctuations are larger and slower for the signal stimulus. In contrast, the STRIPES test was specifically developed for CI users with the focus on avoiding confounding cues by letting subjects identify sweep direction instead of density and by using simple stimuli—to be usable also with newly implanted patients. Here, STRIPES and SMRT were used for evaluating the experimental MAPs and to compare their ability to predict speech perception performance by CI users.

#### Speech Tests

In the speech in quiet (SIQ) test, subjects were presented with sentence lists drawn from the Bamford-Kowal-Bench (BKB; Bench et al. 1979) speech corpus. This consisted of 15 contextual sentences per list with three keywords per sentence, spoken by a British male talker. Subjects were asked to repeat what they heard and were encouraged to guess if unsure about the exact content. The experimenter scored the keywords for each sentence according to the correct answers and the final score per run was calculated by dividing the number of correct keywords by the total number of keywords in that list. Mistakes related to verb tenses or plurality of nouns were deemed correct, but all other mistakes were scored as incorrect.

In the speech in noise (SIN) test, sentence lists from the BKB corpus (as used for SIQ, but different lists) were mixed with time-reversed speech drawn from the Harvard sentences (Rothauser 1969) spoken by a different British male talker. This choice of background noise represented the highly modulated characteristics of competing speech, as it occurs in realistic listening environments, but with the use of an unintelligible masker to avoid informational masking effects (Deeks and Carlyon 2004). An adaptive one-up/one-down procedure with a step size of 2 dB was implemented, to measure the speech reception threshold at which 50 % of the sentences were understood correctly (SRT50; MacLeod and Summerfield 1990). The initial signal-to-noise ratio (SNR) was set to − 4 dB and increased by 2 dB per trial, while repeating a randomly drawn sentence from the list, until the subject recognized the three keywords. The next sentence was then taken from the list and the adaptive procedure started depending on the answer of the subject until all 15 sentences of that list had been presented. A trial was deemed correct if all three keywords were correctly repeated by the subject and the final SRT score for that run was calculated as the average of the last ten SNRs presented.

### Study Protocol

The experiment was organized into five experimental sessions of 2 to 3 h each, which were completed by the subjects on five different days. Electrode impedances were measured at the start of each session using the clinical software. Subjects completed the loudness rating procedure in the first session and the measurement of detection thresholds in the second session (as described in the “Psychophysical Detection Thresholds” section). Before session 3 was performed, the experimental MAPs were constructed by the experimenter (as described in the “Site Selection Based on Polarity Sensitivity” section) and loaded onto the Harmony research processor as pre-defined programming settings. The third and fourth experimental sessions consisted of the evaluation of MAP 1 and MAP 2 using all four evaluation tests. First, three runs of STRIPES were performed for each of the two MAPs, counter-balancing the order of the MAPs across the eight subjects. Second, three runs of SMRT for each MAP were performed using the same order as used for STRIPES. After this, a short break was offered to the subjects and an acclimatization phase was employed to let the subject get used to one of the two experimental MAPs by listening to an audiobook for 15 min (Jules Verne’s *20 Thousand Leagues Under the Sea*, read by a male talker different from the one in the speech tests) and while being able to read along with the printed manuscript. Directly after the completion of the acclimatization phase, their speech recognition in quiet was measured by performing three runs of the test described in “Performance Evaluation.” Following this, the speech recognition in noise test was completed for three runs using the procedure described in “Performance Evaluation.” In the next session, the same procedure was followed, while reversing the order of the MAPs in the STRIPES and SMRT test parts and using the other experimental MAP for the acclimatization part and speech tests. In the final session of the experiment, the third (clinical-like) experimental MAP, MAPC, was tested in all four evaluation tests. The testing followed a similar procedure to the previous sessions by completing three runs of STRIPES, three runs of SMRT, a short break, the acclimatization phase, and three runs for the speech in quiet test followed by three runs of the speech in noise test. In addition, a further set of three runs of STRIPES and SMRT was performed after a short break to obtain the same number of data points for this MAP as for the other two experimental MAPs.

For the evaluation of the two experimental MAPs (MAP 1 and MAP 2), the experiment followed a double-blinded scheme in which neither the experimenter nor the subject knew which experimental MAP was being tested. This was not achieved for the MAP MAPC, which was added as a follow-up measure to compare the performances in MAP 1 and MAP 2 to a reference condition most similar to the subject’s clinical MAP. Here, the subjects but not the experimenter were blinded as to which condition was being tested.

## RESULTS

### Psychophysical Detection Thresholds

**Fig. 2:**
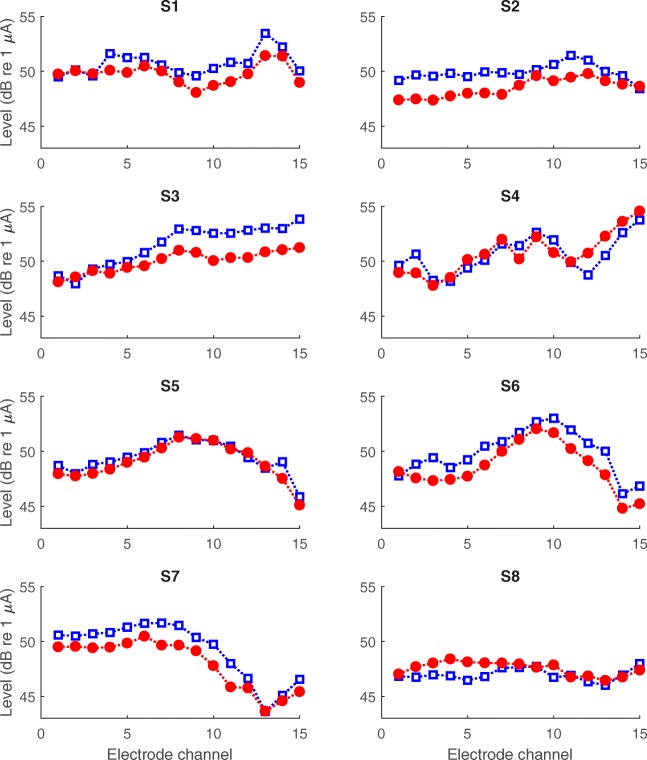
Detection thresholds measured in anodic (CAC, red and filled circles) and cathodic (ACA, blue and open squares) stimulation modes at each electrode site for all subjects

**Fig. 3:**
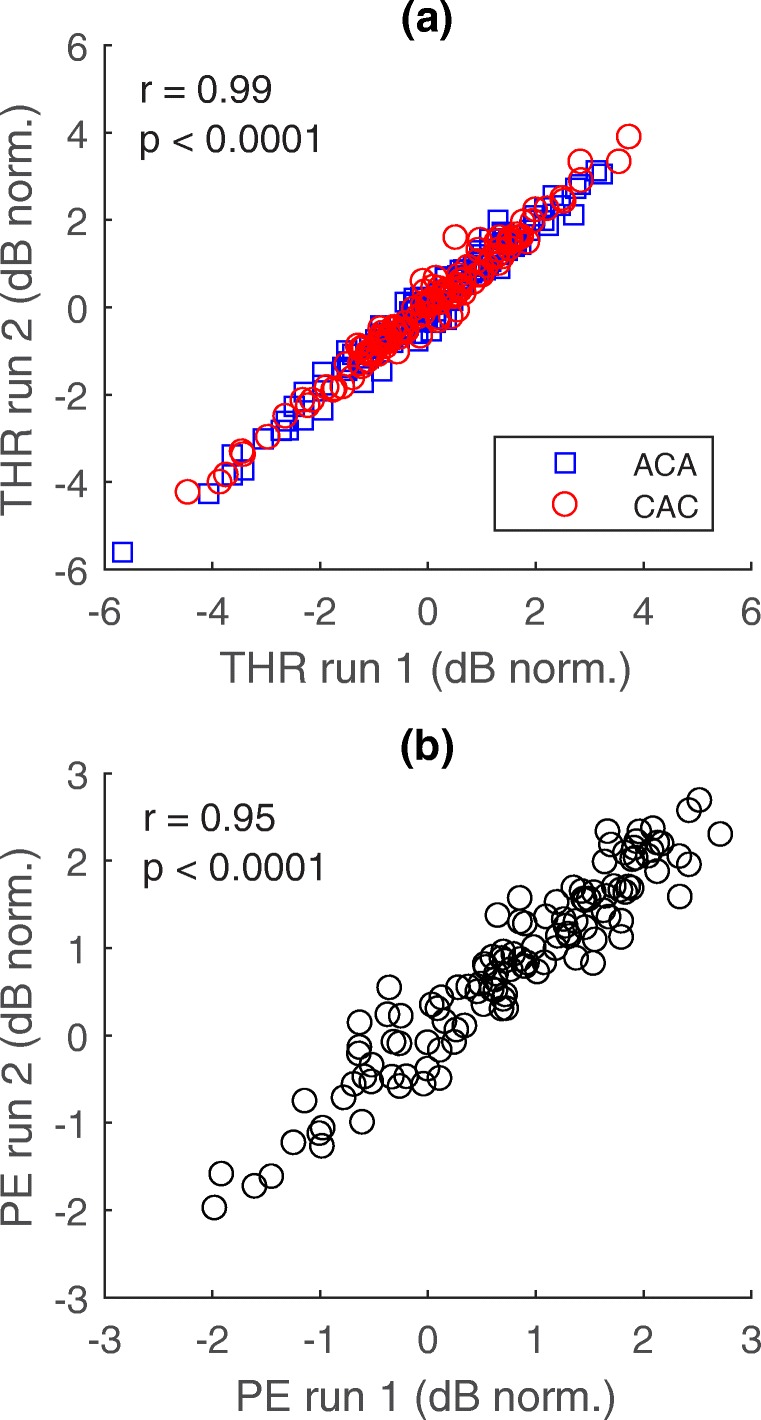
**a** Relationship of the threshold measurements in anodic (CAC, red circles) and cathodic (ACA, blue squares) stimulation mode between the two adaptive tracks performed by all subjects. Thresholds were normalized per subject by subtracting the average threshold across electrodes from each measurement. **b** Relationship of the PE calculated separately for the two adaptive tracks

Detection thresholds measured for both polarities (ACA and CAC) at each electrode site are shown for all subjects in Fig. 2. Note that electrode 16 was excluded from the data analyses, due to this electrode site being active only in two subjects. For the group data, there was a general pattern of lower average thresholds in anodic (CAC, red circles) than in cathodic (ACA, blue squares) stimulation mode for all electrode sites. A paired samples *t* test indicated a significant difference between mean ACA and CAC thresholds [*t*(14) = 13.771, *p* < 0.0001]. A somewhat different shape of the threshold curves depending on the type of electrode array can be observed in Fig. 2, with a significantly larger variability in thresholds across electrode sites for MS (subjects 4, 5, 6, 7) than for 1J (other subjects; Levene’s test, *p* = 0.002). On average, thresholds for the MS array tended to be highest for the middle-numbered electrodes. However, mean thresholds were not different between array types when averaging across electrode sites. The reliability of the threshold tracking procedure was evaluated by calculating the correlation between the two adaptive THR measurements, after normalizing the threshold data for each subject by subtracting, for each data point, the average across electrodes for that subject (Fig. 3a). There were highly significant correlations between the two THR measurements for both polarities ACA and CAC separately (Pearson’s *r* = 0.99, *p* < 0.0001; for both polarities). The average absolute difference in THR measurements between two runs was 0.17 dB with a standard deviation of 0.07 dB.

### Polarity Effect and Experimental MAPs

The PE was based on the average of the thresholds obtained in the two adaptive runs. To evaluate its reliability, we correlated the PE effects based on the first vs second set of measures, after normalizing the data for each subject by subtracting, for each data point, the average for that subject across electrodes. This correlation is shown in Fig. 3b and was highly significant (*r* = 0.95, *p* < 0.0001, *df* = 110). For the data in this study, there was no significant relationship between the average thresholds and the average PE across subjects nor a consistent relationship between the PE and the thresholds across electrodes (ACA, CAC, or clinical thresholds). This latter finding differs from that reported by Carlyon et al. (2018) and is discussed further in the “DISCUSSION” section. The mean and variability in PE were also not different between the two types of electrode arrays used by the subjects.

**Fig. 4:**
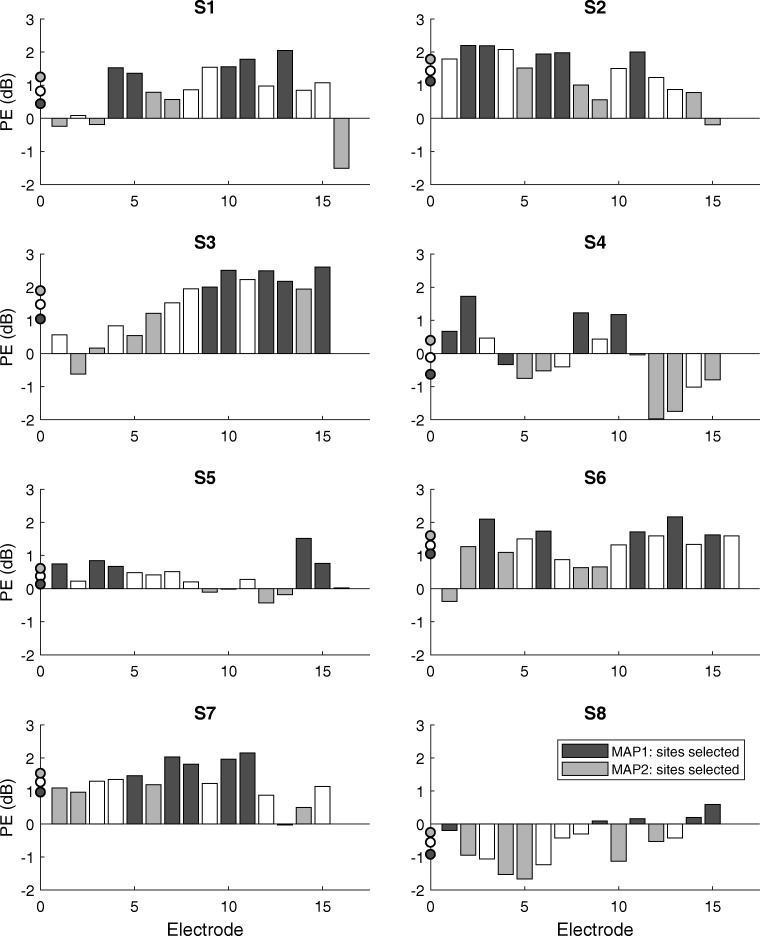
Polarity effect for all subjects and electrode sites measured. The sites that were deactivated in the experimental MAPs are indicated for MAP 1 (black) and MAP 2 (gray). The average PE for all tested electrodes for each subject and for the electrodes remaining in each MAP is indicated on the *y*-axis

The PE is shown for all eight subjects and all measured electrode sites in Fig. 4. The individual patterns of PE values in combination with the site-selection strategy used to construct the two experimental MAPs led to 16 distinct electrode selections. The average PE across electrodes and subjects had a substantial size relative to the electrical dynamic range of the subjects (defined as *M*–*T* level, in dB) of about 36 % on average (with a standard deviation of 20 % and there was an average electrical dynamic range of 3.3 dB across electrodes and subjects). Electrode-wise PE values varied from − 1.98 up to 2.61 dB with subject-wise standard deviations between 0.47 and 1.04 dB. Both the magnitude and variability of the PE across-electrode sites constituted a substantial portion of the electrical dynamic range of the subjects.

**Fig. 5:**
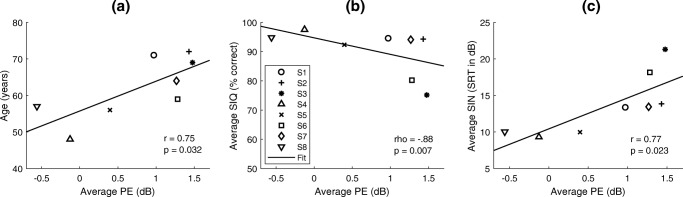
Relationships of the average PE per subject with their age (**a**) and their average speech performance across experimental MAPs in quiet (SIQ, **b**) and in noise (SIN, **c**)

There was a significant positive correlation between the average PE across electrodes with the age of the subjects (*r* = 0.75, *df* = 6, *p* = 0.032; see Fig. 5a) and with the duration of deafness (*r* = 0.76, *df* = 6, *p* = 0.029, not shown). Because age and duration of deafness correlated with each other (*r* = 0.66, *df* = 6, *p* = 0.075), it is unclear which of these factors was responsible for the correlation with PE (partial correlation between PE and deafness duration = 0.76, *p* = 0.029; between PE and age = 0.75, *p* = 0.032).

**Fig. 6:**
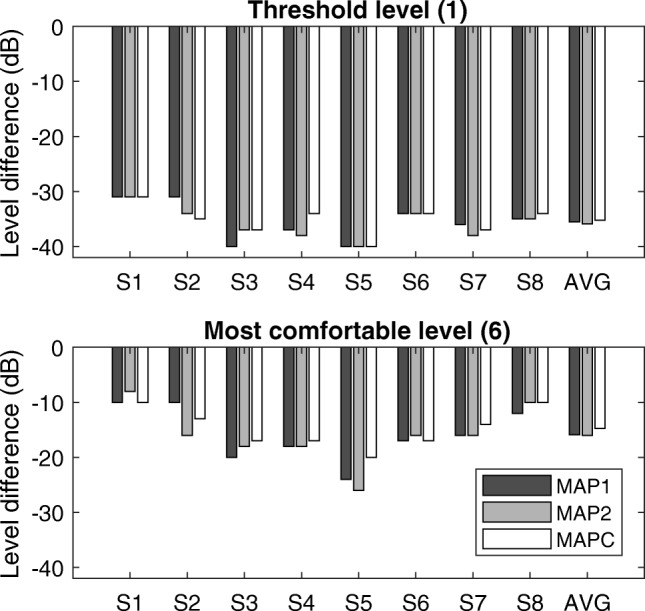
Loudness comparison between the three experimental MAPs at threshold (top) and most comfortable (bottom) level for all subjects individually and on average. The ordinate shows the difference in acoustic level (in dB) relative to the calibrated speech stimuli at 60 dB to achieve loudness category 6 (*M* level) or 1 (*T* level)

The results for the loudness comparison of the three experimental MAPs are shown in Fig. 6. Loudness ratings were very similar between the experimental MAPs and there were no significant differences in the perceived loudness between the three MAPs either at threshold or most comfortable level, as determined by one-way repeated-measures ANOVAs [at THR: *F*(2, 14) = 0.454, *p* = 0.644; at MCL: *F*(2, 14) = 1.232, *p* = 0.321].

### Evaluation Tests

**Fig. 7:**
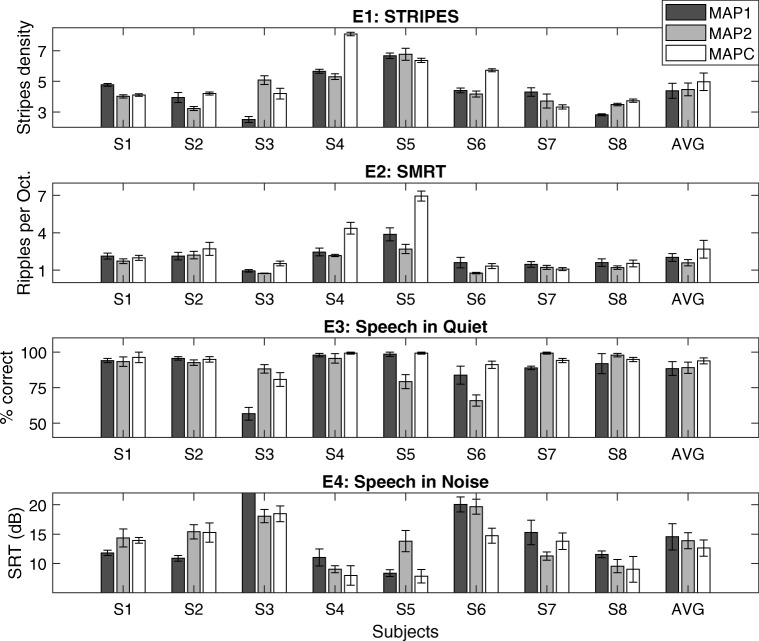
Individual results by all subjects and on average using the three experimental MAPs for all four evaluation tests (E1, STRIPES; E2, SMRT; E3, Speech in Quiet; E4, Speech in Noise). Error bars indicate the standard error of the mean. Asterisks indicate subject-level differences between MAPs (*p* < 0.05). S3 scored 27.4 dB SRT with MAP 1 for E4

The results of the four evaluation tests are shown in Fig. 7 for all subjects. Data are shown for all three MAPs but analyses are initially restricted to MAP 1 and MAP 2. This was done because only those two MAPs were tested in a counterbalanced and double-blind fashion and because our primary outcome measure was the difference in performance between them (with the hypothesis that MAP 1 leads to better performance than MAP 2). These two maps were expected to differ approximately equally from the clinical map, thereby reducing the effect of familiarity on any comparison of the speech scores.

For the non-speech tests, STRIPES and SMRT, test-retest reliability was determined as the correlation between the average performance of MAP 1 and MAP 2 in the three runs in each of the two evaluation test sessions. There were strong and highly significant correlations for both tests (STRIPES: *r* = 0.91, *df* = 6, *p* = 0.0019; SMRT: *r* = 0.87, *df* = 6, *p* = 0.0044). Furthermore, there was no evidence for a practice or fatigue effect; the difference between average scores in sessions 1 and 2 was only − 0.326 for STRIPES and − 0.328 for SMRT, neither of which was statistically significant [STRIPES: *t*(7) = − 1.79, *p* = 0.12; SMRT: *t*(7) = − 1.76, *p* = 0.12]. Of greater importance is the reliability of the differences between the two maps for each test. The subject-wise differences in performance between MAP 1 and MAP 2 were correlated significantly across the two test sessions for STRIPES (*r* = 0.88, *df* = 6, *p* = 0.0042) but not for SMRT (*r* = 0.18, *df* = 6, *p* = 0.68). Hence, we have evidence that the difference in performance between the two MAPs was reliable for the STRIPES test but do not have evidence that this is so for SMRT. That is, for the STRIPES test, a subject who performs better for MAP 1 than for MAP 2 in session 1 will also do so in session 2; we have no evidence that this was the case for SMRT. To evaluate whether the reliability was significantly greater for STRIPES than for SMRT, we compared the two correlations using Fisher’s *r* to *z* transform; this just missed significance (*z* = 1.89, *p* = 0.059, two-tailed).

For the speech tests, the subject-wise normalized speech recognition scores, obtained by subtracting, for each subject, the average performance in the three MAPs from each MAP’s score, in quiet and in noise were strongly correlated (*r* = − 0.83, *df* = 22, *p* < 0.0001), indicating that the differences in speech scores between MAPs were reliable and consistent. Note that for statistical analysis the percentage correct scores for speech in quiet were transformed using the rationalized arcsine transform (RAU; Studebaker 1985). The effect of MAP on performance differed across subjects and across evaluation tests.

The main hypothesis under test was that performance on the evaluation tests would differ significantly between MAP 1 and MAP 2, with better performance for MAP 1. We therefore performed statistical analyses using paired samples *t* tests for those two MAPs. The results showed no significant differences between the experimental MAPs at group level for STRIPES [*t*(7) = − 0.226, *p* = 0.83], SIQ [*t*(7) = − 0.295, *p* = 0.78] and SIN [*t*(7) = 0.555, *p* = 0.59], but there was a small but significant effect for SMRT, whereby thresholds were higher (better) for MAP 1 than for MAP 2 [MAP 1 = 2.03 RPO, MAP 2 = 1.59 RPO, *t*(7) = 3.07, *p* = 0.018]. This significant effect should, we believe, be treated with caution given the fact that, for SMRT, the difference between the two maps did not correlate across the two test sessions. Consistent with the absence of a group-level effect, there were mixed results at subject level, with some subjects showing results consistent with the hypothesis and others against it, both when analyzing all result scores and when restricting the analysis to only statistically significant differences at subject level using paired *t* tests with Bonferroni-Holm correction for eight tests (one for each subject). For the latter case, there were three subject-level differences significant between MAP 1 and MAP 2 for the STRIPES test (S1, S3, S8) and one for the SIN test (S3), out of which just S1 showed an effect in the predicted direction with STRIPES.

We additionally performed some analyses while including the data for MAPC. This revealed no significant effect of MAP on any of the outcome measures, as assessed by one-way repeated-measures ANOVAs for each outcome measure. No significant overall differences were found [STRIPES: *F*(2, 14) = 1.153, *p* = 0.344; SIQ: *F*(2, 14) = 0.905, *p* = 0.427; SIN: *F*(2, 14) = 0.994, *p* = 0.395; SMRT: *F*(1.132, 7.923) = 3.857, *p* = 0.083, *df* for SMRT adjusted using Huynh-Feldt correction due to sphericity violation]. Although our comparisons between the experimental maps revealed no significant group-level differences for any outcome measure, there were significant subject-level differences between the experimental MAPs and MAPC (tested using two-tailed, paired *t* tests with Bonferroni-Holm correction for 16 tests, with 2 per subject) for the STRIPES and SMRT tests. For the STRIPES test, six subjects showed a benefit of MAPC over one of the experimental maps, one (S7) showed no significant differences, and S1 showed a significant subject-level benefit with MAP 1 over MAPC. For the SMRT test, one subject (S5) showed a significant advantage for MAPC over MAP 2. For SIQ, most subjects scored highly with all three experimental MAPs (> 80 % correct, apart from S3 with MAP 1 and S6 with MAP 2) and there were no significant subject-level differences between the experimental MAPs and MAPC. For SIN, there was a large variability in performance between subjects, with SRT scores ranging from 7.8 dB up to 20.7 dB (excluding the very high SRT for S3 using MAP 1), and there were again no significant subject-level differences. Note that for SIQ and SIN, there were only three runs averaged per score compared to STRIPES and SMRT with six runs per score, which made it less likely to detect statistical differences at subject level for the speech tests.

We also examined whether either of the spectro-temporal measures predicts, for a given subject, which MAP will produce the best speech perception. If so, this would provide preliminary evidence that the spectro-temporal test could be used clinically in order to identify the processing strategy that will provide the best possible speech perception for a given subject. To perform the evaluation, we normalized all measures to the mean across the three MAPs for each subject and then correlated these normalized values. This is mathematically equivalent to the method recommended by Bland and Altman (1995). For the STRIPES test, this revealed correlations that were in the predicted direction for both speech tests; the correlation was significant for SIN (*r* = − 0.59, *df* = 14, *p* = 0.016) and just missed significance for SIQ (*r* = 0.48, *df* = 14, *p* = 0.059). For SMRT, the correlation was not significant for SIN (*r* = − 0.33, *df* = 14, *p* = 0.21) and just missed significance for SIQ (*r* = 0.48, *df* = 14, *p* = 0.059).

While there was no significant relationship between the average detection thresholds or the variance in thresholds across electrodes and the normalized evaluation test scores, there was a significant across-subject correlation between the PE averaged across electrodes and both the SIQ and SIN scores averaged across MAPs per subject (SIQ: not normally distributed as tested with Lilliefors test, *p* = 0.001, Spearman’s rho = − 0.88, *df* = 6, *p* = 0.0072; SIN: *r* = 0.77, *df* = 6, *p* = 0.023), with better performance associated with lower PE (see Fig. 5b, c). Because PE is a difference score, these across-subject correlations are unlikely to be driven by cognitive differences between subjects. They are consistent with, but do not prove, the idea that subjects having low PEs exhibit good neural health and good speech perception. Furthermore, there were strong associations between the duration of deafness and speech perception in quiet (*r* = − 0.91, *df* = 6, *p* = 0.002) and in noise (*r* = 0.95, *df* = 6, *p* < 0.001) consistent with results reported in previous studies (van Dijk et al. 1999; Holden et al. 2013; Plant et al. 2016). However, there were no significant relationships between the duration of deafness and the spectro-temporal tests (STRIPES: *r* = − 0.55, *df* = 6, *p* = 0.158; SMRT: *r* = − 0.65, *df* = 6, *p* = 0.081).

## DISCUSSION

### Comparison Between MAPs

Stimulus detection thresholds were measured in a group of CI users for triphasic, low-rate stimuli in both anodic and cathodic polarities to calculate the PE, the difference in polarity sensitivity at threshold level, for all active electrode sites. The PE demonstrated subject-specific patterns that were distinct from the thresholds per se and the clinical thresholds based on high-rate, biphasic stimulation. PE values showed strong test-retest reliability and were substantial in size relative to the electrical dynamic range of the subjects.

The proposed site-selection strategy was evaluated using two experimental MAPs that were constructed by deactivating the electrode sites with either the five highest or five lowest PE values for each subject. The two MAPs, MAP 1 and MAP 2, were used in four listening tests to evaluate performance differences and to compare against a third MAP, MAPC, most similar to the subjects’ clinical MAP. Statistical analysis revealed no significant differences between MAP 1 and MAP 2 for any of the evaluation tests at group level, except for a small (and, as we have argued above, potentially unreliable) advantage for MAP 1 in the SMRT test. No significant differences were observed when all three maps were analyzed together. Hence, the site-selection strategy was not successful in improving the overall listening performance for this group of CI users. While the small amount of acclimatization provided here made a performance benefit of the reduced-electrode MAPs over MAPC unlikely, this cannot explain why performance was not better for MAP 1 than for MAP 2, as both of these maps were unfamiliar to the subjects. Nevertheless, it is of some interest that performance was also not significantly worse overall with the reduced-electrode MAPs, indicating a strong robustness of CI users to changes in their spectral mapping strategy. This was most obvious for speech in quiet, where all subjects performed at very high levels with all or at least two of the three MAPs under test. Thus, MAPC was never clearly better than the better of the channel-reduced MAPs in the speech tests at subject level. As expected, variability in between-subject performance and within-subject differences between MAPs were more prominent for the speech-in-noise test than in the speech-in-quiet test. There was no clear pattern at group level, but results indicate that there were significant differences in listening performance between the experimental MAPs (MAP 1 and MAP 2) at subject level with the STRIPES test. This shows that spectro-temporal processing, as measured by a non-speech test, can in principle be affected by the choice of which electrodes to disable.

### Comparison with Previous Studies on Site-Selection Strategies

Several studies have shown significant effects of site-selection strategies on listening performance in CI users either at group level (Garadat et al. 2012, 2013; Saleh et al. 2013; Zhou 2017) or at subject level (Noble et al. 2013, 2014; Bierer and Litvak 2016). Differences between studies included the measures upon which electrode sites were deactivated, the number of electrodes deactivated, the rules used for site selection, and the coding strategy employed for the presentation to the subjects. Those studies that reported significant group-level effects over the clinical MAP (Zhou 2017; Garadat et al. 2013), used a site-selection strategy that deactivated a small proportion of electrodes (5/22), that were evenly distributed across five regions along the electrode array, and used the ACE coding strategy. Both of those studies compared the experimental MAP directly to the clinical MAP without using, for all subjects, a control condition that differed from the clinical MAP by a similar amount as did the experimental MAP. Interestingly, they demonstrated significant improvements in speech recognition even without acclimatization to the experimental MAP, a promising outcome for a new processing strategy in CI users. However, one could argue that the reported benefits were due to decreased between-electrode interactions due simply to having fewer remaining active electrodes. This argument cannot explain the results of a previous study (Garadat et al. 2012) that reported substantially better speech-in-noise perception when using the electrode sites (10/22) with the lowest modulation detection thresholds (MDTs) compared to a condition with the 10 sites having the highest MDTs. That study used the same subjects as in Garadat et al. (2013) but with a different site-selection method and a CIS signal-processing strategy. Taken together, the findings of Garadat et al. (2012, 2013) provide support for a successful site-selection strategy based on modulation detection thresholds, but differences in methodology complicate the direct comparison of results. In addition, as Bierer et al. (2015b) have argued, the differences in MDTs observed in those studies may have been mediated by differences in loudness.

The methodology by Bierer and Litvak (2016) was most similar to the current study and compared two experimental MAPs to a MAP with all channels active as in the clinical MAP. While there was no significant effect at group level, Bierer and Litvak reported beneficial effects for both experimental MAPs for some subjects, especially for those subjects with poorer speech perception performance. This trend was not observed in the current study, in which the two subjects with the poorest speech performance (S3 and S6) did not obtain improvements with the reduced-channel MAPs over the all-channel MAP MAPC. Finally, it is worth noting that, unlike the experiments reported here, the majority of previous studies have not used double-blind procedures to evaluate the different experimental strategies. This may lead to unconscious biases both on the part of the subject and the experimenter. Placebo effects are ubiquitous in medical research and we advise that beneficial effects of site selection or other novel programming methods should be confirmed using double-blind procedures before being used to inform clinical practice.

### Spectro-Temporal Tests and Their Prediction of Speech Scores

The spectro-temporal tests, STRIPES and SMRT, showed strong test-retest reliability for average performance between MAPs, and the difference between the two MAPs on the STRIPES test was consistent across sessions. Furthermore, STRIPES successfully predicted the variation in speech-in-noise scores across MAPs, once between-subject differences were removed. The results found in this study support the potential applicability of the STRIPES test to predict differences between subject-specific speech-in-noise scores obtained with different MAPs. We did not find this evidence for the SMRT test, possibly because it may have been too difficult for most subjects or because of confounding cues introduced by the stimuli. In contrast to STRIPES, the SMRT test failed to produce consistent subject-wise differences between MAP 1 and MAP 2 across testing sessions, indicating that scores were not reliable. In comparison, STRIPES’ reliability likely resulted from its easier procedure, developed specifically for CI users, and by avoiding confounding cues that are not related to spectro-temporal processing. One of the main advantages of non-speech tests over conventional speech tests is that no acclimatization period is needed for subjects to learn the relationship between a novel pattern of stimulation and the identity of speech segments, as is the case for speech tests (Davis et al. 2005). It is worth noting that we evaluated speech perception after only 15 min of acclimatization; the rationale underlying STRIPES is that it should predict the pattern of performance across MAPs once acclimatization is complete. If so, then it is possible, although of course not certain, that even stronger correlations would have been obtained had we used longer acclimatization periods.

### Polarity Effect as an Estimate of Neural Health

The absence of a group-level effect for the site-selection strategy used here does not negate the PE as an estimate of local neural health. There were several factors due to the electrode deactivation that may have interacted with and changed the performance in the evaluation tests such as spectral shifts, spectro-temporal distortions, and changes in stimulation sites. All of these alterations may require longer periods of acclimatization than provided in this study. Furthermore, it is still not known whether the selective use of neural regions with better neural health leads to improved speech perception. In support of the rationale for the PE as an estimate of neural health, there were significant correlations for the average PE with the age and the duration of deafness of the subjects and strong associations between the PE and the average speech perception performance in quiet and in noise. The PE was lower for younger subjects, those with shorter deafness durations, and for the ones who performed better on the speech scores. However, our small sample size means that these correlations should be interpreted with caution, and previously reported relationships between speech performance and demographic factors such as duration of deafness were found to account for less variability than reported here (Holden et al. 2013; Plant et al. 2016). Furthermore, the correlations between PE and other measures might depend on the subset of subjects tested. For example, the variation in speech scores among a group of subjects who differed strongly in cognitive ability might be dominated by those cognitive factors and therefore correlate only weakly with the PE. The point that correlations may depend on the subjects tested is also relevant to a discrepancy between the finding that, across electrodes, the PE correlated significantly with the average of the anodic and cathodic thresholds in the study of Carlyon et al. (2018) but not here. It may be that for some subjects, the across-electrode variation in average threshold is dominated by factors other than neural health, such as the electrode-modiolar distance (EMD; Long et al. 2014; DeVries et al. 2016), whereas the PE is sensitive to neural health but less to EMD than the thresholds per se (Mesnildrey 2017). A test of this hypothesis would be to collect thresholds from a large number of subjects from whom there are postoperative CT scans, split these into groups with large vs small across-electrode variations in EMD, and measure the correlations between the PE and average thresholds in the two groups.

Future investigations are needed to evaluate subject-specific measures of spatial selectivity and electrode interaction for potential relationships with the PE patterns and the site-selection strategy outcomes in this study. Furthermore, electrode-specific measurements of electrically evoked compound action potentials (Undurraga et al. 2010; Spitzer and Hughes 2017; Hughes et al. 2018) or the auditory change complex (Mathew et al. 2017) could serve as objective measures of neural functioning along the electrode array to validate the PE measurements and to inform the site-selection strategy (Prado-Guitierrez et al. 2006; Ramekers et al. 2015).

## CONCLUSIONS

We evaluated a site-selection strategy, based on polarity sensitivity at threshold level, designed to improve speech perception by CI users. Eight subjects completed four evaluation tests, two spectro-temporal tests and two speech recognition tests, with three experimental MAPs, one of which was most similar to their clinical MAP. The other two experimental MAPs were constructed by deactivating the five electrodes with the best or worst local neural health as estimated from the polarity sensitivity measure, PE. The data measured to construct and evaluate the experimental MAPs showed strong test-retest reliability. Results revealed no significant differences between the experimental MAPs at group level, but there were significant differences between the MAPs at subject level. These individual differences in outcomes are in line with the previous pattern of findings in CI users that show a large variability in performance and in the benefits of novel strategies (Zwolan et al. 1997; Noble et al. 2014; Bierer and Litvak 2016; Zhou 2016). The STRIPES test, but not the SMRT test, was successful in predicting the differences in speech-in-noise scores between the experimental MAPs and may prove useful for clinical and research applications to predict the effect of novel programming strategies on speech perception on a patient-by-patient basis.

The measure of polarity sensitivity, PE, was related to the age, the duration of deafness, and to the speech perception performance of the CI users, consistent with the hypothesis that polarity sensitivity reflects the neural health in the cochlea (Mesnildrey 2017; Carlyon et al. 2018). However, the absence of a clear performance advantage for any of the experimental MAPs underlines the need for further investigations into the appropriate metric for site-selection strategies.
